# PD-1 inhibitor-induced systemic sclerosis with early interstitial lung disease and autoimmune thyroiditis in advanced gastric cancer: a case report and literature review

**DOI:** 10.3389/fonc.2026.1776228

**Published:** 2026-03-23

**Authors:** Qi Lyu, Xiquan Xu, Wanyue She, Jueying Wang, Maidina Aimurula, Meng Zhou, Jingjing Ding, Yu Gu

**Affiliations:** 1 Department of Oncology, Affiliated Hospital of Nanjing University of Chinese Medicine, Nanjing, China; 2 First Clinical Medical College, Nanjing University of Chinese Medicine, Nanjing, China

**Keywords:** autoimmune thyroiditis, immune checkpoint inhibitor, interstitial lung disease, PD-1, systemic sclerosis

## Abstract

Immune checkpoint inhibitors (ICIs) have revolutionized anti-tumor therapy, yet their use is increasingly complicated by immune-related adverse events (irAEs). We present a rare case in which sequential administration of two PD−1 inhibitors (ICIs), Camrelizumab and Sintilimab, in combination with conventional chemotherapy led to ICI-related systemic sclerosis (ICI-SSc) with early-stage interstitial lung disease (ILD) and autoimmune thyroiditis (AIT). The clinical presentation also suggested a possible scleroderma overlap syndrome. The underlying pathology is likely linked to widespread immune activation following PD−1 pathway blockade. Early identification of ICI-SSc is crucial. Once the diagnosis is confirmed, the suspected drug should be discontinued immediately, and a personalized treatment plan should be initiated, ideally with consideration of the safety profile of steroid-based regimens. Furthermore, our findings raise important considerations regarding pharmacovigilance in the context of sequential ICIs use.

## 1 Introduction

Systemic sclerosis (SSc; scleroderma) is a rare connective tissue disorder characterized by fibrosis and represents the rheumatic disease with the highest mortality (1, 2). Its multi-system involvement affects the skin and multiple organs with the pattern and severity of organ damage determining prognosis (2, 3). Interstitial lung disease (ILD) occurs in 35–52% of SSc patients and is a leading cause of death (4). Progressive ILD combined with pulmonary arterial hypertension (PAH) confers particularly poor outcomes, especially in diffuse cutaneous SSc (dcSSc) (1). Clinical or subclinical hypothyroidism is uncommon in SSc, though more prevalent among female patients even in iodine-deficient regions (5). Concurrent hyperthyroidism generally indicates worse prognosis (5). SSc complicated with autoimmune thyroiditis (AIT) is even rarer (6). To date, no clinical reports describe SSc presenting simultaneously with ILD and AIT. While the pathophysiological mechanisms of SSc remain unclear, anti-tumor drugs have been classified as the leading drug-related triggers of SSc (7). Immune checkpoint inhibitors related SSc (ICI-SSc) is a rare immune-related adverse event (irAE) with an incidence of less than 1% but with a very high mortality rate (8). Due to the lack of foundational studies on the related mechanisms, our knowledge of ICI-SSc remains reliant on case reports, and clinical management is guided by prior experiential summaries.

Here, we present for the first time a multidisciplinary discussion (MDT) case of a patient with advanced gastric cancer who developed SSc with early-stage ILD and autoimmune thyroiditis after sequential ICIs combined with conventional chemotherapy. We also explore the potential mechanisms and clinical status of ICI-SSc based on literature review.

## 2 Case presentation

### 2.1 Anamnesis

**Figure 1:**
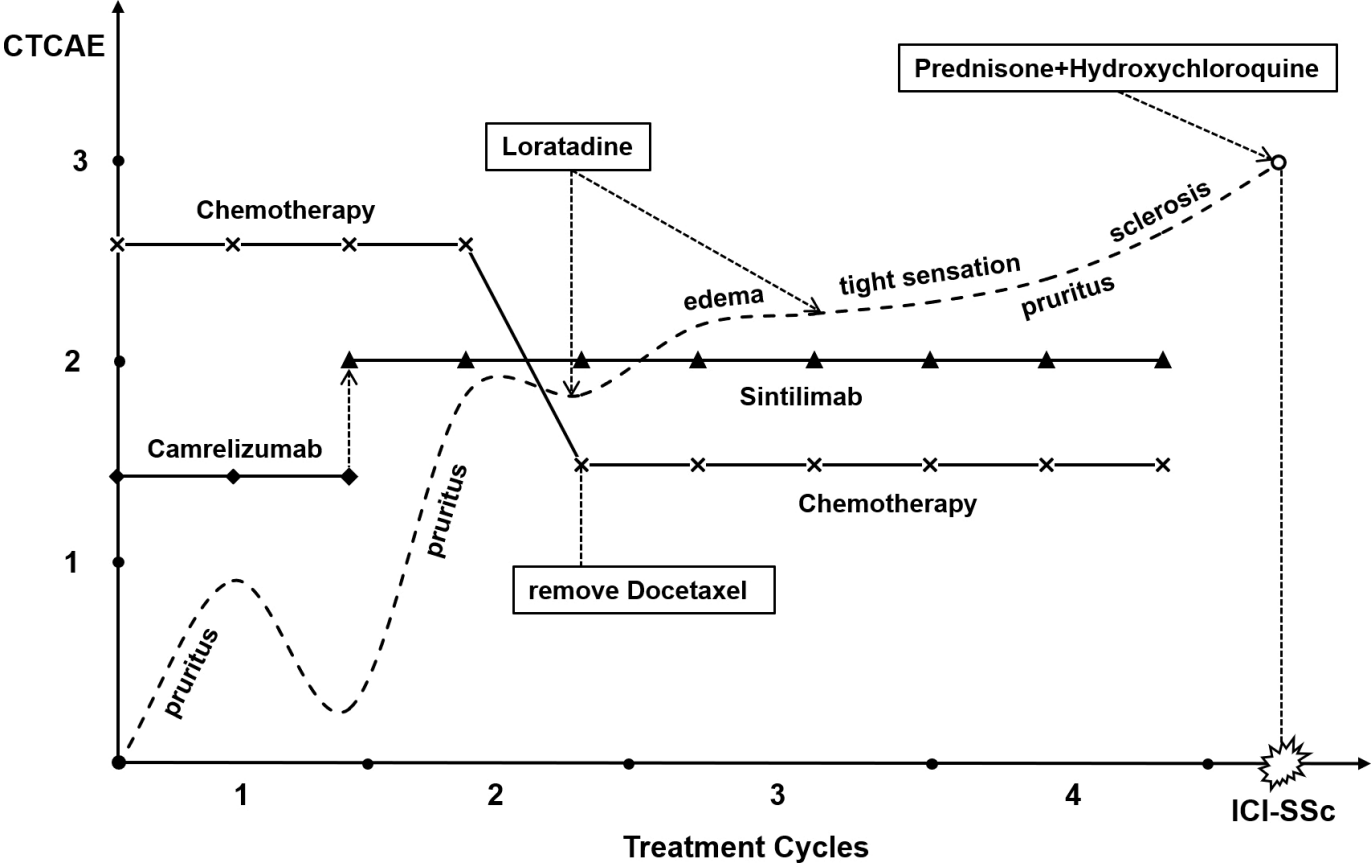
Timeline diagram.

In 2024, a 61-year-old woman was diagnosed with poorly differentiated adenocarcinoma of the gastroesophageal junction and gastric fundus, with lymph node metastasis. The patient had a prior history of diabetes, which she currently manages with subcutaneous insulin injections for blood glucose control. Baseline assessments indicated stable blood glucose levels, and no other underlying diseases were found. In July 2024, the patient underwent her first cycle of anti-tumor treatment: Camrelizumab (a recombinant human anti-PD-1 monoclonal antibody) (200mg iv, d0), Oxaliplatin (100mg iv, d1), Docetaxel (180mg iv, d1), and Tegafur, Gimeracil and Oteracil Potassium(S-1) (40mg po, twice daily, d1-d14). On the third day after completing intravenous infusion, the patient developed generalized pruritus, considered to be a Grade 1 (Common Terminology Criteria for Adverse Events, CTCAE 5.0) irAE caused by Camrelizumab. After the patient’s pruritus improved, a transition to a presumably safer immunotherapy regimen was initiated during the second cycle of anti-tumor treatment in August 2024. The regimen consisted of Sintilimab [a recombinant human anti-PD-1 monoclonal antibody (9)] (200mg iv, d0), with the original chemotherapy regimen. However, the patient’s tolerance remained poor. She experienced severe pruritus all over her body, excoriation and ulceration of the skin after scratching, edema in both forearms, and a tight sensation throughout her skin. The irAE level reached Grade 2 (CTCAE 5.0). Loratadine was administered during treatment to manage pruritus as recommended by the guidelines. In September and October 2024, docetaxel was omitted from both treatment cycles to minimize its potential contribution to adverse effects. The regimen was adjusted to Sintilimab (200mg iv d0) in combination with Oxaliplatin (100mg iv d1) and S-1 (40mg po, twice daily, d1-d14). After September 2024, the patient’s pruritus improved, but the tightness of the entire skin continued to worsen. The tightest areas involved the backs of the hands and waist, with marked hardening of the skin on both hands, forearms, and the shins. This severely affected the patient’s daily life, with the irAE at one point approaching Grade 3 severity (CTCAE 5.0). After completing four cycles of immunotherapy combined with chemotherapy, the patient voluntarily discontinued anti-tumor treatment because of severe skin sclerosis. (**Figure 1**: Timeline Diagram.)

### 2.2 Diagnosis and treatment

**Figure 2:**
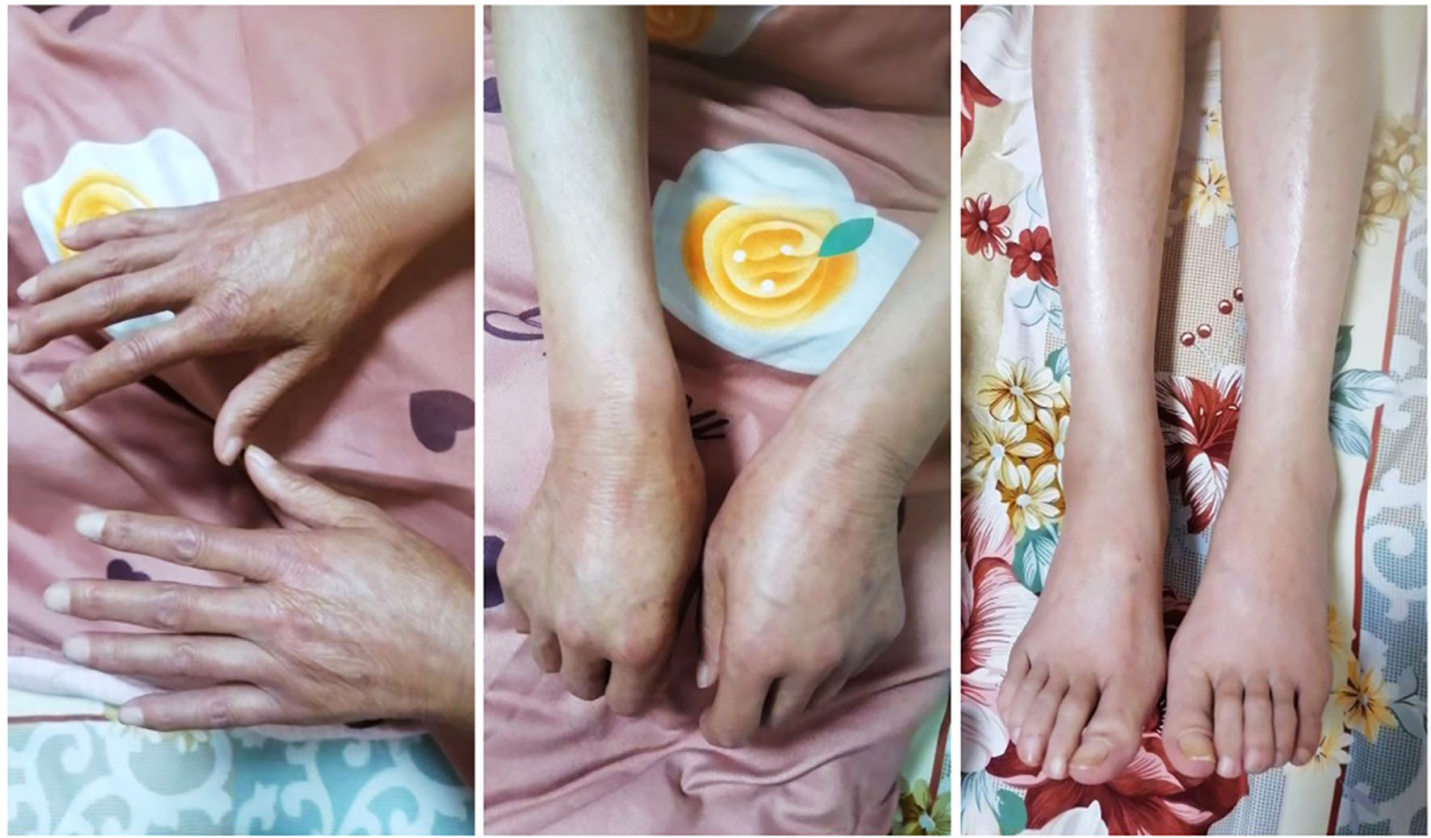
Scleroderma presentation in January 2025.

**Figure 3:**
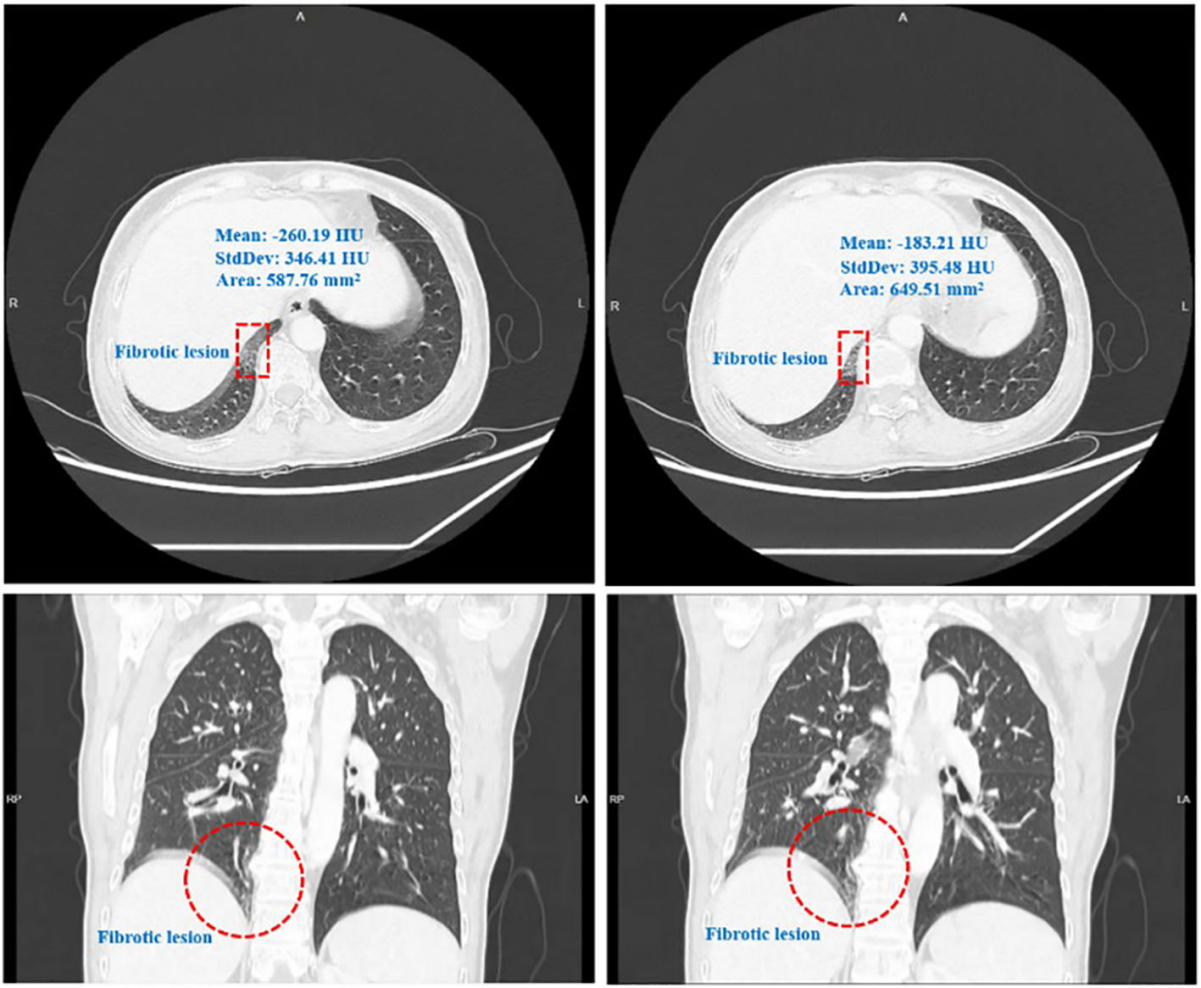
Early ILD CT imaging features.

In January 2025, the patient presented at the hospital due to skin sclerosis and pruritus. Chief complaints included: a sensation of tightness throughout the body, shallow facial wrinkles, thickened and hardened skin in multiple areas (more severe in both forearms and lower limbs), generalized itching and dryness, inability to make a fist, lower limb edema, and dry mouth. Physical examination revealed thickened, hardened, and shiny skin on both hands, forearms, and the shins; the local skin could not be pinched; there was restricted movement of the finger joints, metacarpophalangeal, and wrist joints; both hands could not make a complete fist; fingers were swollen; both lower limbs showed pitting edema; facial expression appeared rigid, but there was no obvious skin hyperpigmentation; no suspicious subcutaneous sclerotic nodules were found; there was no joint pain nor significant Raynaud’s phenomenon. (**Figure 2**: Scleroderma Presentation In January 2025.) Laboratory tests revealed positive antinuclear antibody (ANA), elevated immunoglobulin G (IgG), elevated erythrocyte sedimentation rate (ESR), positive Anti-Ro-52, and concurrent AIT [elevated Anti-thyroid peroxidase antibody (TPOAb), elevated Anti-thyroglobulin antibody (TgAb)]. (**Table 1**: Laboratory Test Results and Reference Values.) Chest CT showed interstitial changes adjacent to the spine in the right lower lobe, with a reticular pattern visible. (**Figure 3**: Early ILD CT Imaging Features.) The patient declined both skin/gland biopsy and pulmonary function testing (PFT), citing financial constraints and a fear of invasive procedures.

**Table 1:** Laboratory test results and reference values.

Inspection item	Result	Reference value
C reactive protein (CRP)	3.26	<8mg/L
Red blood cell count	2.79	3.8~5.10×^12/L
Hemoglobin (Hb)	99	115~150g/L
Eosinophil percentage (EOS%)	15.6	0.4~0.8%
Absolute eosinophil count (AEC)	0.65	0.02~0.52×^9/L
Erythrocyte sedimentation rate (ESR)	36	<20mm/h
Hemoglobin A1c (HbA1c)	5.5	4~6%
Thyroid-stimulating hormone (TSH)	10.1	0.27~4.2uIU/mL
Free T3	3.4	2~4.4pg/mL
Free T4	0.7	0.93~1.7ng/dL
Anti-thyroglobulin antibody (TgAb)	252	<115 IU/mL
Anti-thyroid peroxidase antibody (TPOAb)	96.9	<34 IU/mL
Immunoglobulin G (IgG)	20.1	7.51~15.6g/L
Immunoglobulin A (IgA)	2.11	0.82~4.53g/L
Immunoglobulin M (IgM)	1.18	0.46~3.04g/L
Antinuclear antibody (ANA)	Positive(1:320)	<1:100
ANA pattern	Cytoplasmic fibrous pattern+Nucleolar pattern	–
Anti-Ro-52	Positive(+)	Negative
Anti-centromere antibody (ACA)	Negative(-)	Negative
Anti-nRNP/Sm	Negative(-)	Negative
Anti-ds-DNA	Negative(-)	Negative
Anti-Jo-1	Negative(-)	Negative
Anti-SSA	Negative(-)	Negative
Anti-SSB	Negative(-)	Negative
Anti-Scl-70	Negative(-)	Negative
Cytoplasmic antineutrophil cytoplasmic antibody (cANCA)	Negative(-)	Negative
Perinuclear antineutrophil cytoplasmic antibody, formalin-resistant (p-ANCA, formalin-resistant)	Negative(-)	Negative
Perinuclear antineutrophil cytoplasmic antibody, formalin-sensitive (p-ANCA, formalin-sensitive)	Negative(-)	Negative
Anti-Proteinase 3 antibody (Anti-PR3)	<2.0	<20AU/mL
Anti-Myeloperoxidase antibody (Anti-MPO)	6	<20AU/mL
Anti-Glomerular Basement Membrane antibody (Anti-GBM)	<2.0	<20AU/mL

After a multidisciplinary team consultation among dermatology, rheumatology, and oncology, this case was determined to meet The 2013 ACR/EULAR Criteria for the Classification of Systemic Sclerosis (10). The modified Rodnan skin score (mRSS) (11) was used to assess the degree of skin sclerosis, with a final score of 26 out of 51. Based on the Naranjo adverse drug reaction assessment scale (score of 9) and the WHO-UMC causality criteria, this case was determined to be an adverse drug reaction (ADR) attributable to PD-1 inhibitors therapy. After excluding primary systemic sclerosis and other scleroderma-like disorders, the patient was ultimately diagnosed with ICI-SSc complicated by ILD and AIT, with an overall irAE severity graded as level 3 (CTCAE 5.0). To alleviate the patient’s pruritus, we administered dexamethasone (5mg iv, qd) combined with loratadine (10mg po, qn) for antihistamine therapy from admission. After the pruritus improved, the treatment regimen was switched to prednisone acetate tablets (5mg po, qd) combined with hydroxychloroquine sulfate tablets (0.2g po, bid), with concurrent acid suppression for gastric protection and calcium supplementation.

### 2.3 Follow-up

**Figure 4:**
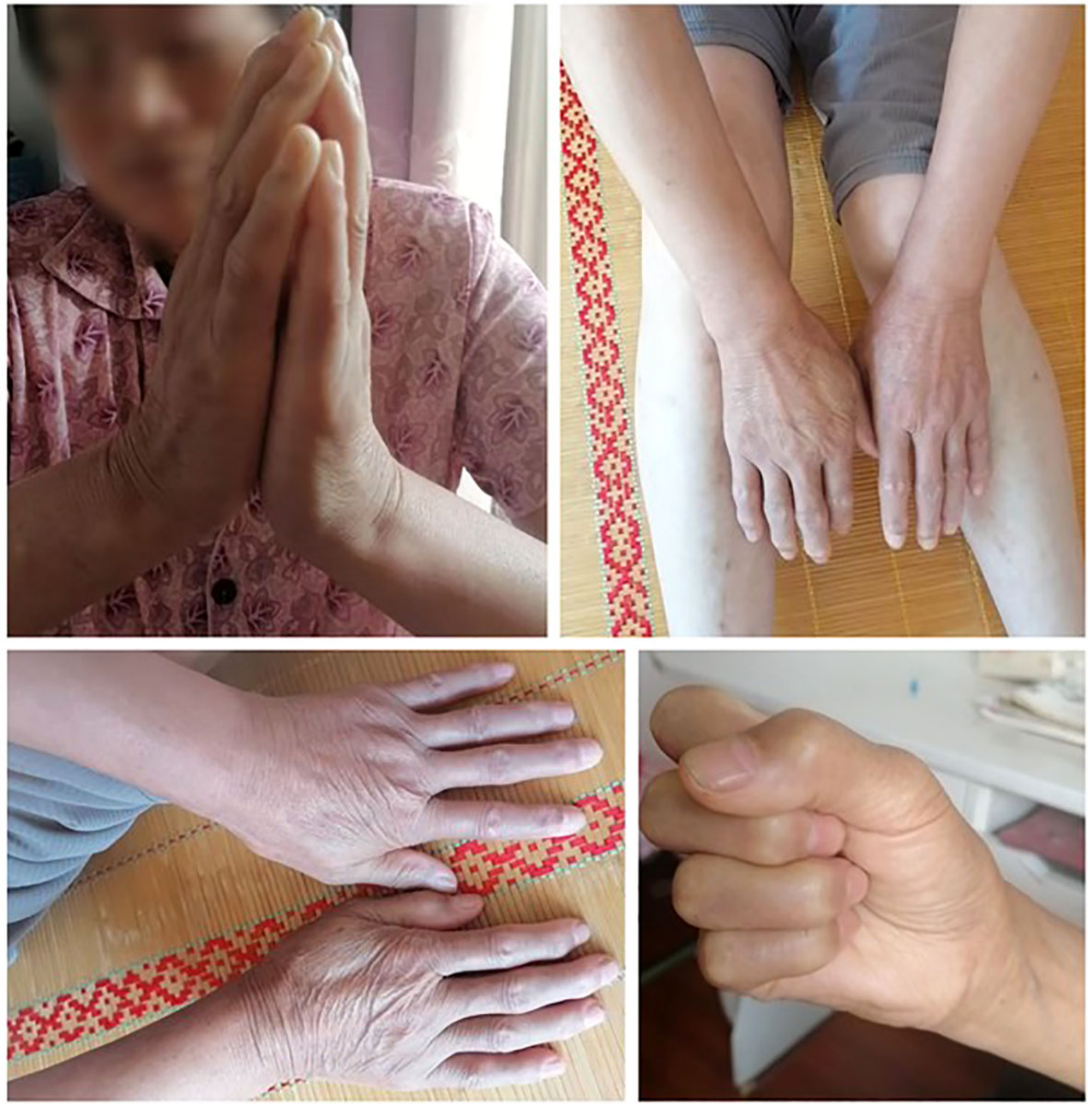
Follow-up pictures on July 15, 2025.

In March 2025, upon follow-up, we found that the patient was still taking prednisolone acetate and hydroxychloroquine sulfate tablets. Pruritus had virtually disappeared, and the skin tightness and sclerosis had basically resolved, with her mRSS score maintained at 20 points. In July 2025, we performed a second follow-up. The patient had discontinued corticosteroids on her own in May, reporting significant improvement in skin sclerosis and that the sensation of skin tightness had disappeared; these symptoms no longer interfered with her daily life, though she continued to experience some limitation in finger joint movement. (**Figure 4**: Follow-up Pictures.) The patient reported experiencing mild wheezing upon exertion, such as after climbing stairs, which was not accompanied by any other respiratory symptoms. Unfortunately, the patient had already abandoned anti-tumor treatment, and her tumor progression status remains unknown.

## 3 Discussion

**Figure 5:**
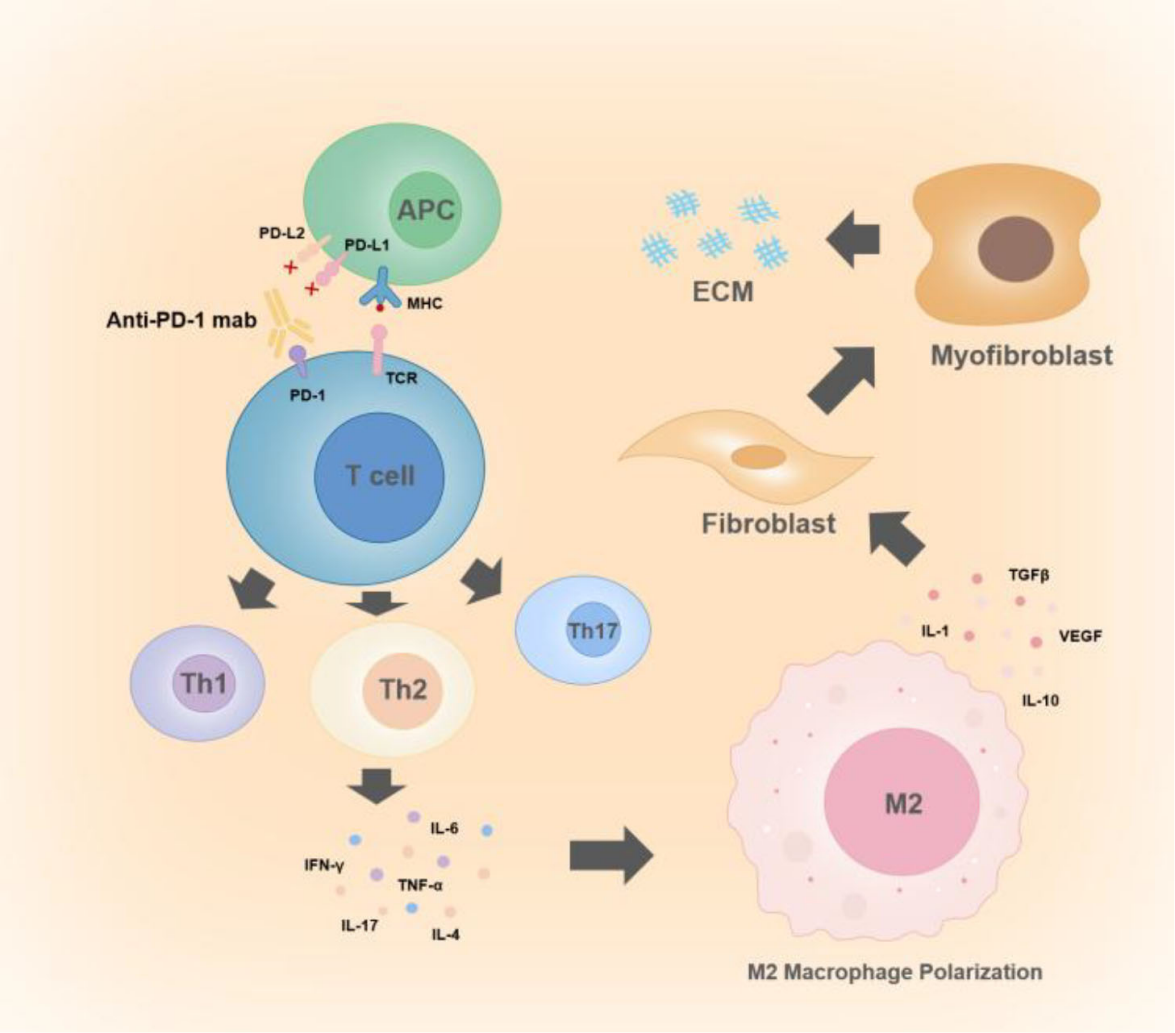
Mechanism of PD-1 inhibitors induced SSc. PD-1 inhibitors(Anti-PD-1 mabs) bind to PD-1 receptors on T cells, thereby blocking the PD1-PDL1/2 pathway and leading to the inhibition of negative co-stimulatory signals. Concurrently, antigen−presenting cells(APC) engage T cells via some interactions such as MHC-TCR and CD28-CD80/86, activating positive co−stimulatory pathways. The inhibition of negative and activation of positive co-stimulation promote the development of T cells into T helper cell subsets (Th1, Th2, Th17). These subsets secrete cytokines (IFN-γ, TNF-α, IL-4, IL-6, IL-17) that stimulate M2 macrophage polarization, which is the core mechanism in the fibrotic process of SSc. Polarized M2 macrophages in turn produce profibrotic mediators including TGF−β, VEGF, IL−1, and IL−10, which promote the differentiation of fibroblasts into myofibroblasts, leading to fibrosis. Fibroblasts secrete large amounts of components such as collagen types I and III, fibronectin, and proteoglycans, resulting in extracellular matrix(ECM) deposition and further exacerbating fibrosis.

This study presents a case of sequential PD-1 inhibitors combined with conventional chemotherapy resulting in multiple irAEs, including ICI-SSc and AIT, along with early stage ILD. While SSc is recognized as an immune-mediated rheumatic condition, its exact pathological mechanisms have yet to be fully clarified. Current research is mainly focused on immune activation, microvascular lesions, and the accumulation of extracellular matrix (12, 13). Many anti-tumor agents have been identified as triggers for SSc (7). Among these, reports of SSc induced by PD-1 inhibitors in ICIs have shown a disproportionate increase, particularly with Nivolumab and Pembrolizumab, whereas CTLA-4 inhibitors have not shown a similar trend (14). This observation implies that the PD-1 signaling axis plays a distinct role in the pathogenesis of SSc. Studies have shown that the PD-1/PD-L1 pathway is negatively correlated with a variety of autoimmune diseases (15). From a pathophysiological perspective, the mechanisms underlying PD-1 inhibitor-induced SSc may involve the following: blockade of the PD-1/PD-L1 pathway disrupts peripheral immune tolerance, leading to impaired regulatory T cell (Treg) function and reduced control over effector T cells, alongside concurrent activation of co-stimulatory pathways. This cascade promotes the development of T cells into Th1, Th2, and Th17 cells, ultimately establishing a Th2-dominant immune response (14). These three T cell subsets are major contributors to SSc fibrosis and are central to the overlapping mechanisms of SSc and AIT (16–18). The resulting immune imbalance drives aberrant fibroblast activation and their differentiation into myofibroblasts, which in turn secrete excessive extracellular matrix components. These deposits accumulate in the skin and internal organs, culminating in irreversible fibrosis (18). Notably, the variable incidence of SSc induced by different ICIs may reflect their distinct modes of action on T cells. PD-1 and PD-L1 inhibitors primarily exert their effects in peripheral tissues and are involved in the effector phase of the immune response. By disrupting peripheral immune tolerance, they may directly contribute to fibrosis processes and the development of SSc (15). (**Figure 5**: Mechanism of PD-1 Inhibitors Induced SSc.) In contrast, CTLA-4 inhibitors primarily target central lymphoid organs and are more involved in the early stages of immune activation. Their influence on specific autoimmune responses is relatively indirect, which may explain the lower observed risk of SSc associated with CTLA-4 inhibitors (15, 16).

In the diagnosis of ICI-SSc, it is critical to distinguish it from primary SSc and other scleroderma-like disorders. This study approaches the differential diagnosis from three perspectives: clinical manifestations, serological features, and disease outcomes. Primary SSc is usually defined as an autoimmune disease characterized by microangiopathy, immune activation, and widespread fibrosis (1). Beyond the fibrosis of the skin and viscera, its diagnosis mainly relies on the presence of specific autoantibodies (ACA, anti-Scl-70, PR3) and microvascular involvement manifestations (such as Raynaud’s phenomenon, nailfold capillary abnormalities) (18). In contrast, ICI-SSc exhibits a distinct clinical phenotype. Although its fibrotic features can resemble those of primary SSc, microvascular involvement is less commonly observed in ICI-SSc. The incidence of Raynaud’s phenomenon is comparatively low, and serum SSc-specific autoantibodies (ACA, anti-Scl-70, and anti-PR3) as well as ANA tend to be absent (18, 19). On the other hand, Scleroderma-like lesions induced by chemotherapeutic agents, particularly taxanes, represent another important consideration in the differential diagnosis of ICI-SSc. Similar to ICI-SSc, these Scleroderma-like lesions generally lack vascular involvement and serological abnormalities, aside from a clear history of exposure. However, their temporal evolution and outcomes differ. Such lesions are most often localized to the lower extremities and classically progress from an edematous to a sclerotic phase (20, 21). Importantly, they tend to regress rapidly upon withdrawal of the offending agent (21). By contrast, the trajectory of ICI-SSc is notably different. Discontinuation of ICIs alone is frequently insufficient to arrest fibrotic progression. And it frequently necessitates intervention with glucocorticoids such as prednisone (14). Furthermore, given the well- established association between SSc and malignancy (15), paraneoplastic SSc must also be considered. The timing of diagnosis offers a critical clue. Paraneoplastic SSc is typically identified concurrently with the malignancy, follows a course that parallels tumor burden, and may regress with successful cancer treatment (7). Finally, clinicians should remain alert to scleroderma mimics that may confound the diagnosis. Eosinophilic fasciitis typically presents with symmetrical pain and induration of the extremities, accompanied by marked peripheral eosinophilia. It usually spares the fingers and is not associated with Raynaud’s phenomenon. Scleredema is characterized by firm, nonpitting edema of the neck, shoulders, and upper trunk, with a doughy texture and no acral involvement. Scleromyxedema is often associated with IgG-λ paraproteinemia and widespread lichenoid papules. Localized scleroderma (morphea) involves circumscribed patches of skin fibrosis without systemic involvement or microvascular changes (1, 18). In the case we report, the patient presented with classic features of cutaneous sclerosis and visceral involvement, along with consistent serological findings and a clear history of immunotherapy. After excluding primary SSc and other scleroderma-like disorders, the clinical presentation met the diagnostic criteria for ICI-SSc.

If SSc patients also suffer from another rheumatic immune disease, it is called SSc overlap syndrome (22). Studies suggest that among individuals with diffuse cutaneous SSc (dcSSc), those who are ANA-positive and ACA-positive are more likely to develop overlap syndromes. In particular, the presence of Ro antibodies carries a nearly 30% risk of coexisting Sjögren’s syndrome (SS) (23). In the case, the patient experienced notable dry mouth symptoms. Laboratory testing revealed positivity for both ANA and Ro-52 antibodies, raising the possibility that this may represent an ICIs-induced overlap syndrome(SSc/SS). Given that ICIs trigger broad immune activation, patients that experience one irAE are more likely to experience more irAEs. Our patient was no exception. But the coexistence of multiple irAEs, especially when ICI-SSc is involved, remains quite rare. By using the following keyword combinations: (“immune checkpoint inhibitor” OR “PD-1” OR “Nivolumab” OR “Pembrolizumab” OR “Sintilimab” OR “Camrelizumab”) AND (“scleroderma” OR “systemic sclerosis”), a PubMed review of the past decade turned up only four cases of ICI-SSc accompanied by other irAEs (24–27). Among them, one was a fatal case involving an unspecified ICI (24), and another described overlap syndrome induced by nivolumab (26). Most of these reports involved Pembrolizumab or Nivolumab (24). To date, no cases have been published linking Camrelizumab or Sintilimab to multiple irAEs. Our report is the first to describe ICI-SSc with additional irAEs following sequential use of two PD-1 inhibitors. Based on analysis of European pharmacovigilance data, the majority of patients with ICI-SSc generally experience improvement following therapeutic intervention and tend to have a positive clinical outcomes (24). Nevertheless, the prognosis becomes considerably worse once visceral complications develop or other irAEs emerge alongside ICI-SSc (2–6). In more severe cases, ICI-SSc may trigger scleroderma renal crisis or coincide with ICIs-associated nephritis, potentially leading to life-threatening Grade 4 (CTCAE 5.0) adverse events (14, 24).

It is also noteworthy that, the patient rechallenged a different PD-1 inhibitor after achieving remission from the initial irAE. This sequential administration of two PD-1 inhibitors constitutes what is termed in pharmacovigilance as an exposure switch (28). Both Camrelizumab and Sintilimab are PD-1 inhibitors with similar pharmacological mechanisms. Theoretically, the toxicity and risk associated with sequential use should not differ substantially from those of continued monotherapy with a single agent (28). Any differences in safety may instead be attributable to distinct pharmacokinetic properties. Available evidence indicates that rechallenging patients with a PD-1 inhibitor after an irAE carries a recurrence rate of approximately 34.2% for any grade irAEs and around 11.7% for high-grade events. These rates are not significantly different from those observed during initial treatment (29, 30). That said, high-quality prospective evidence supporting the safety and validity of this rechallenge strategy remains limited. Since all PD-1 inhibitors disrupt peripheral immune tolerance through the same pathway, a like-for-like substitution does little to mitigate the risk of sustained immune activation. For this reason, current clinical guidelines recommend permanent discontinuation of ICIs for serious adverse events exceeding grade 3 (CTCAE 5.0) (31, 32). Rechallenge is considered only in select cases (colitis, low-grade events and complete resolution, no effective alternative therapy) (28). From a pharmacovigilance perspective, rare events involving high-risk re-exposure, as illustrated in this case, underscore the need for continued real world data collection and heightened clinical vigilance. They also serve as a reminder for clinicians to weigh the risks carefully when considering sequential ICIs therapy.

Managing ICI-SSc poses substantial challenges, as it requires coordinated attention to irAEs, underlying malignancies, SSc activity, and the complications. From the perspective of irAE management, current guidelines recommend that in cases when it exceeds grade exceeds 3 (CTCAE 5.0), ICIs should be discontinued promptly and high-dose corticosteroids, or even combined immunosuppressive therapy, should be initiated (31, 32). However, in ICI-SSc, systemic corticosteroids carry a risk of triggering scleroderma renal crisis, particularly in dcSSc patients (14, 33). According to current guidelines and expert committee recommendations, it is advisable to use the lowest possible dose of oral steroids to control SSc progression, with a daily cumulative dose not exceeding the prednisone equivalent of 15mg and regular renal function monitoring (13, 33). In addition to corticosteroids, hydroxychloroquine and methotrexate are first-line treatments for SSc. Hydroxychloroquine combined with low-dose glucocorticoids is used both in SSc and Sjögren’s syndrome management (34). For dcSSc with ILD, methotrexate (MTX) has already been replaced by mycophenolate mofetil (MMF) (35). The anti-IL6 monoclonal antibody Tocilizumab represents a promising therapeutic avenue for ICI-SSc (18). At present, Tocilizumab is approved not only for SSc-ILD, but also for steroid-refractory irAEs, such as inflammatory arthritis (36). More importantly, owing to the cascade of immunological activation, discontinuation of therapy alone is insufficient to control the further progression of ICI-SSc (14). In cases of high mortality rates and limited treatment options, early identification and diagnosis of ICI-SSc become particularly critical.

In this case report, we present a patient who developed ICI-SSc with early ILD and AIT following sequential treatment with two PD-1 inhibitors. Through a rigorous combination therapy approach, the patient’s condition was stabilized and his quality of life significantly improved. Admittedly, this study has several limitations. Due to the patient’s refusal to undergo invasive procedures, the diagnosis was established through a comprehensive assessment that respected his preferences. This included a thorough physical examination revealing typical extensive cutaneous sclerosis, serological findings (positive ANA, elevated IgG and ESR, positive anti-Ro-52 antibodies, elevated TPOAb and TgAb), and imaging features suggestive of early ILD. The diagnosis was further supported by current classification criteria and clinical scoring systems. Although a labial gland biopsy was not performed, we suspect this patient may represent a case of SSc overlap syndrome with Sjögren’s syndrome, given the presence of dry mouth symptoms, antibody positivity (ANA, anti-Ro-52), and the statistical probability of SS occurrence in such contexts. During follow-up, the patient reported exertional dyspnea and decreased exercise tolerance. However, due to financial constraints and the patient’s unwillingness to undergo PFT, we were unable to quantify the extent of lung volume reduction or perform adequate risk stratification. This leaves open the possibility of silent disease progression and delayed therapeutic intervention. Given the uncertainty surrounding the long-term prognosis of ILD in this patient, extended follow-up will be essential.

## 4 Conclusion

We present a rare case of ICI-SSc with multiple comorbidities occurring after sequential PD-1 inhibitor therapy. The pharmacovigilance issues associated with rechallenge strategies involving sequential ICIs warrant close attention. In the context of limited treatment options, a regimen combining low-dose corticosteroids and antirheumatic drugs appears to be a promising strategy for treating ICI-SSc. More importantly, early recognition of ICI-SSc is critical. Once scleroderma-like skin reactions occur, ICIs should be discontinued immediately, relevant examinations should be performed as thoroughly as possible, and individualized interventions should be initiated as early as possible to prevent potentially fatal outcomes. Although reports of ICI-SSc with multiple complications remain rare, current data indicate that the PD-1 pathway may be closely associated with the underlying mechanisms of ICI-SSc and other irAEs, warranting further research.

## Data Availability

The original contributions presented in the study are included in the article/Supplementary Material. Further inquiries can be directed to the corresponding author.
